# Probiotic Therapy in Patients with Nonalcoholic Steatohepatitis in Zagazig University Hospitals

**DOI:** 10.5005/jp-journals-10018-1226

**Published:** 2017-05-05

**Authors:** Sameh M Abdel Monem

**Affiliations:** Department of Tropical Medicine, Faculty of Medicine, Zagazig University, Zagazig, Egypt

**Keywords:** Nonalcoholic fatty liver disease, Probiotics, Steatohepatitis.

## Abstract

**Aim:**

Nonalcoholic fatty liver disease (NAFLD) is probably the most common liver disorder in the world. A subgroup of NAFLD patients is characterized by injury to the hepatocytes and inflammation in addition to excessive fat (steatohepatitis), the latter condition is nominated nonalcoholic steatohepatitis (NASH). This work aimed to evaluate the role of probiotics on the outcome of NASH in patients admitted to the Tropical Medicine Department, Faculty of Medicine, Zagazig University (inpatients and outpatients).

**Materials and methods:**

This study was performed on 30 patients (17 males and 13 females), with body mass index from 30 to 35 and average age of 44 years with bright fatty liver in ultrasonography and raised alanine transaminase (ALT) and aspartate transaminase (AST) and positive liver biopsy findings. The patients were divided into group I (case group) that included 15 patients who received probiotics and group II of 15 patients as control group who did not receive probiotics; the study was conducted between November 2014 and April 2016. Clinical assessment, laboratory evaluation, pelvic-abdominal ultrasound, and liver biopsy of all cases were carried out.

**Results:**

In this study, there was significant decrease in liver enzymes (ALT and AST) and no statistically significant other laboratory findings. Also there was relief for dyspepsia in some patients.

**Conclusion:**

Probiotics treatment is effective, safe, well-tolerated, inexpensive, appropriate for long-term use, and optimally, works at multiple levels to downregulate inflammatory mediators, and therefore, probiotics could be an option in the treatment of NASH.

**How to cite this article:** Monem SMA. Probiotic Therapy in Patients with Nonalcoholic Steatohepatitis in Zagazig University Hospitals. Euroasian J Hepato-Gastroenterol 2017;7(1):101-106.

## INTRODUCTION

Nonalcoholic fatty liver disease (NAFLD) is a state defined by extreme fat accumulation in the form of triglycerides (steatosis) in the liver (more than 5% of liver cells histo-logically). It is considered nowadays as one of the most frequent causes of abnormal liver tests all over the world, and it ranges from simple steatosis to advanced fibrosis and cirrhosis. A subgroup of NAFLD patients were characterized by injury to the hepatocytes and inflammation in addition to excessive fat (steatohepatitis). The latter condition, nominated nonalcoholic steatohepatitis (NASH), is practically indistinguishable histologically from alcoholic steatohepatitis, and it is characterized by fatty infiltration of the liver with different degrees of inflammation, necrosis, and fibrosis, almost identical to those of alcoholic liver disease; without significant alcohol ingestion.^1^

Simple steatosis seen in NAFLD does not correspond to increased short-term morbidity or mortality, but advancement of this condition to NASH noticeably increases the risks of fibrosis, cirrhosis (cryptogenic cirrhosis), liver failure, and hepatocellular carcinoma (HCC). The prevalence of NAFLD ranges from 10 to 24% of the general population, while NASH affects about 3% of the lean population and nearly half of morbidly obese people.^2^

The term nonalcoholic steatohepatitis or NASH is expressing the clinical and pathological characters of NAFLD coupled with the pathological characters mostly present in alcoholic liver disease itself. This description is still proper as NAFLD can progress to cirrhosis with fat from simple steatosis, through NASH and fibrosis.^3^

Nonalcoholic steatohepatitis is a chronic liver disease, i.e., earning increasing significance due to its great prevalence all over the world, the difficulty in diagnosis with noninvasive diagnostic methods, and the risk of succession to advanced fibrosis and cirrhosis, all of which make NASH difficult for doctors. Although the natural history of NASH and the causes of disease progression remain mostly unknown, more than a few data suggest that in some patients the disease may follow an indolent course until it progresses to end-stage liver disease.^4,5^

Most patients have the clinical characteristics of insulin resistance syndrome, including obesity, hypertension, glucose intolerance, and typical dyslipidemia. Insulin resistance plays a role not only in obese individuals but also in lean nondiabetic patients with hepatic steatosis.^6^

The "two-hit" hypothesis is the leading theory for the pathogenesis of NASH. This model suggests that the initial event or first "hit" leads to steatosis, and the second "hit" leads to necroinflammation and fibrosis. Insulin resistance has been implicated as the first "hit" and various insults, such as oxidative stress, cytokine effects, and fatty acid toxicity are suspected as potential other hits that lead to hepatocellular injury.^7^ Tumor necrosis factor-alpha (TNF-α) in the liver can contribute to oxidative stress; furthermore, it may contribute to insulin resistance.^8^ Additionally, TNF-α also initiates fibrosis both by direct activation of hepatic stellate cells and by stimulating production of tumor growth factor (TGF)-beta, a potent profibrogenic cytokine.^9^

A complex relationship exists between endotoxins, stellate cell activation, and release of cytokines and chemokines due to increased intestinal bacterial overgrowth, increased gut permeability, and reduced endotoxin scavenging by the reticuloendothelial system. The endotoxin releases a battery of cytokines; interleukin (IL)-1, IL-6, and TNF-α from nonparenchymal cells.^10^ The general belief that intestinal bacterial overgrowth has a role in NAFLD is supported by the evidence that obesity and diabetes, which are the major risk factors for NASH, are also associated with intestinal dysmotility^11^ and small bowel bacterial overgrowth.^12^ Probiotics are traditionally defined as viable microorganisms that have beneficial effects in the prevention and treatment of specific pathologic conditions when they are ingested.^13^ There are many proposed mechanisms by which probiotics may protect the host from intestinal disorders, including production of inhibitory substances, blocking of adhesion sites, competition for nutrients, degradation of toxin receptor, and stimulation of immunity.^13^ Probiotics affect intestinal bacterial flora by increase of anaerobic bacteria and decrease of the population of potentially pathogenic microorganisms.^14^

This work aimed to evaluate the role of probiotics on the outcome of NASH in patients admitted to the Tropical Medicine Department, Faculty of Medicine, Zagazig University (inpatients and outpatients).

## MATERIALS AND METHODS

This randomized controlled study was performed on 30 patients (17 males and 13 females), with body mass index (BMI) from 30 to 35 and average age of 44 with bright fatty liver in ultrasonography and raised alanine transaminase (ALT) and aspartate transaminase (AST) and positive liver biopsy findings. Patients were divided into group I (case group) that included 15 patients who received probiotics and group II that included 15 patients (control group) who did not receive probiotics and were consulting at Tropical Medicine Department, Faculty of Medicine, Zagazig University Hospitals between November 2014 and April 2016.

### Inclusion Criteria

Patients with elevated liver enzymes and bright liver by ultrasonography were examined and investigated for selection of patients with NASH, which was proved by liver biopsy.

### Exclusion Criteria

Patients with history of significant alcohol consumption more than 20 gm/day or under dietetic regimen; patients with positive hepatitis B and C virus markers; patients who were taking lipid-lowering medications, metformin, or thiazolidinediones; and patients with any other metabolic liver disease were excluded from the study. Kidney function tests were performed: For exclusion of cases with renal impairment. Compensated and decompensated cirrhotic patients; hepatic focal lesion; AST/ALT ratio >1 were also excluded.

All patients were subjected to the following: Full clinical history with stress on age and sex, change in appetite, dyspepsia, abdominal distension, right hypochondrial pain, and fatigue. Patients with history of medical diseases, e.g., diabetes mellitus, hypertension, bilharziasis, chronic viral hepatitis, pituitary disease, adrenal disease or pancreatic disease, history of surgical operation, e.g., gastric bypass or jejunoileal bypass were also excluded.

All patients were checked for parameters of general examinations, such as blood pressure, pulse, face complexion, hand and lower limb examination, body weight, height, and BMI that was defined by Kumar and Clark.^15^

Local examination of abdomen and liver was accomplished.

Laboratory assessment included complete blood count, hemoglobin level, white blood cells, and platelets counts. Parameters of liver function tests were also checked. Imaging of abdomen and the liver was done.

### Probiotic Therapy

The patients were kept on probiotic supplementation. Acidophilus capsule *(Lactobacillus acidophilus,* which contains 2 billion viable organism, and mixture of rice flour, gelatin, and magnesium stearate) was given to patients 30 minutes before meal three times daily for 1 month duration (Acidophilus, Swanson Health Products, USA website, www.swansonvitamins.com).

### Statistical Analysis

Data were checked, entered, and analyzed using Statistical Package for the Social Sciences version 18 for Windows. Data were expressed as mean ± standard deviation for quantitative variable, number and percentage for qualitative one. Chi-squared (χ^2^) or t-test and paired t-test were used when appropriate; p < 0.05 was considered significant; p < 0.001 was considered highly significant.

## RESULTS AND DISCUSSION

Nonalcoholic fatty liver disease is probably the most common liver disorder in the world.^16^ It is generally believed that NAFLD affects 2.8 to 24% of the general population and affects adults and children.^17^

In addition, NAFLD is the consequence of excess triglyceride accumulation in hepatocytes in the absence of significant alcohol consumption.^18^ It includes a spectrum of hepatic changes from steatosis alone, to NASH, fibrosis, cirrhosis, and even HCC.^19^

Probiotics are traditionally defined as viable microorganisms that have beneficial effects in the prevention and treatment of specific pathologic conditions when they are ingested.^13^ There are many proposed mechanisms by which probiotics may protect the host from intestinal disorders, including production of inhibitory substances, blocking of adhesion sites, competition for nutrients, degradation of toxin receptor, and stimulation of immunity.^13^ Probiotics affect intestinal bacterial flora by increase of anaerobic bacteria and decrease of the population of potentially pathogenic microorganisms.^14^

This study is aimed to evaluate the role of probiotics on the outcome of NASH in humans.

This randomized controlled study was performed on 30 patients (17 males and 13 females), with BMI from 30 to 35 and average age of 44 with bright fatty liver in ultrasonography and raised ALT and AST and positive liver biopsy findings. Patients were divided into group I (case group) that included 15 patients who received probiotics and group II that included 15 patients (control group) who did not receive probiotics and were consulting at Tropical Medicine Department, Faculty of Medicine, Zagazig University Hospitals between November 2014 and April 2016.

In the present study of demographic, clinical, and abdominal ultrasound (U/S) findings before treatment for both groups, no significant difference was seen. Also this study showed no significant difference as regards complete blood count, liver function test, kidney function test, prothrombin time (PT), international normalized ratio (INR), lipid profile, and blood sugar. Due to all patients were complaining from nonalcoholic steatohepatitis then were divided randomly into two groups.

In the present study, patients were matched with control subject as regards posttreatment biochemical findings, which showed no significant difference (Tables 1 and 2). But there was a highly significant difference in ALT and significant difference in AST. Otherwise, other biochemical changes showed no significant difference between both groups This is in agreement with the results of Solga and Diehl,^20^ Loguercio et al,^21^ and Portincasa et al,^22^ which found similar changes in ALT and AST. These findings denote that the use of probiotics in these patients plays some role in improving the necroinflam-matory status of the liver (Tables 3 to 6).

**Table 1:** **Table 1:** Comparison between groups I and II as regards demographic, clinical, and U/S findings

*Variables*		*Group I (n = 15)*		*Group II (n = 15)*		*p-value*	
Age (years)		44.20 ± 5.51		44.33 ± 5.62		0.948	
Gender							
Male		9 (60%)		8 (53.3%)		0.713	
Female		6 (40%)		7 (46.7%)			
BMI (kg/m^2^)		32.56 ± 1.19		33.05 ± 1.27		0.284	
Clinical presentation							
Asymptomatic		8 (53.3%)		15 (100%)		0.006	
Dyspepsia		4 (26.7%)		0 (0%)		0.100	
Fatigue		2 (13.3%)		0 (0%)		0.483	
Right hypochondria!		1 (6.7%)		0 (0%)		1.000	
pain							
Clinical examination							
Hepatomegaly		4 (26.7%)		4 (26.7%)		1.000	
Splenomegaly		0 (0%)		0 (0%)		1.000	
Ascites		0 (0%)		0 (0%)		1.000	
Liver in U/S							
Average		4 (26.7%)		5 (33.3%)		0.709	
Enlarged		11 (73.3%)		10 (66.7%)			
Spleen in U/S							
Average		15 (100%)		15 (100%)		1.000	
Splenomegaly		0 (0%)		0 (0%)			
Ascites in U/S							
Absent		15 (100%)		15 (100%)		1.000	
Present		0 (0%)		0 (0%)			

**Table 2:** **Table 2:** Comparison between groups I and II as regards laboratory findings before treatment

*Variables*		*Group I before (n = 15)*		*Group II before (n = 15)*		*p-value*	
Hemoglobin (g/dL)		13.16 ± 0.88		13.57 ± 0.84		0.208	
WBCs (× 10^3^/mm^3^)		6.78 ± 1.72		7.36 ± 1.85		0.383	
Platelet count (× 10^3^/mm^3^)		300.60 ± 78.33		312.20 ± 75.56		0.683	
Protein (g/dL)		7.47 ± 0.77		7.45 ± 0.89		0.819	
Albumin (g/dL)		4.60 ± 0.44		4.64 ± 0.45		0.835	
TSB (mg/dL)		0.88 ± 0.38		0.86 ± 0.41		0.950	
DSB (mg/dL)		0.11 ± 0.04		0.12 ± 0.05		0.665	
ALT (U/L)		81.45 ± 23.32		83.53 ± 12.01		0.962	
AST (U/L)		44.05 ± 14.65		42.73 ± 9.95		0.967	
PT (sec)		15.19 ± 1.72		15.86 ± 1.94		0.325	
INR		1.00 ± 0.09		1.04 ± 0.10		0.336	
Serum creatinine (mg/dL)		0.90 ± 0.29		1.05 ± 0.30		0.186	
BUN (mg/dL)		18.06 ± 6.81		19.13 ± 7.46		0.686	
Triglycerides (mg/dL)		257.80 ± 55.02		245.33 ± 70.78		0.755	
Cholesterol (mg/dL)		258.60 ± 31.70		250.53 ± 45.12		0.771	
LDL (mg/dL)		158.53 ± 23.67		154.73 ± 33.24		0.721	
HDL (mg/dL)		48.73 ± 4.83		48.80 ± 5.00		0.971	
FBS (mg/dL)		104.86 ± 21.33		107.06 ± 27.06		0.807	
PBS (mg/dL)		161.13 ± 53.37		173.06 ± 73.91		0.819	

WBC: While blood cell; TSB: Total serum bilirubin; DSB: Direct serum bilirubin; BUN: Blood urea nitrogen; LDL: Low-density lipoprotein; HDL: High-density lipoprotein; FBS: Fasting blood sugar; PBS: Peripheral blood smear

**Table 3:** **Table 3:** Comparison between groups I and II as regards laboratory findings after treatment

*Variables*		*Group I after (n = 15)*		*Group II after (n = 15)*		*p-value*	
Hemoglobin (g/dL)		13.20 ± 0.85		13.67 ± 0.71		0.108	
WBCs (× 10^3^/mm^3^)		6.79 ± 1.70		7.36 ± 1.67		0.366	
Platelet count (× 10^3^/mm^3^)		298.40 ± 76.37		309.26 ± 70.02		0.688	
Protein (g/dL)		7.47 ± 0.77		7.42 ± 0.80		0.857	
Albumin (g/dL)		4.58 ± 0.45		4.66 ± 0.41		0.493	
TSB (mg/dL)		0.87 ± 0.37		0.88 ± 0.37		0.967	
DSB (mg/dL)		0.11 ± 0.04		0.12 ± 0.05		0.613	
ALT (U/L)		46.10 ± 20.75		82.53 ± 10.41		<0.001	
AST (U/L)		38.20 ± 11.58		55.86 ± 5.11		0.03	
Serum creatinine (mg/dL)		0.89 ± 0.26		0.91 ± 0.20		0.142	
BUN (mg/dL)		17.73 ± 6.13		18.46 ± 6.45		0.752	

WBC: While blood cell; TSB: Total serum bilirubin; DSB: Direct serum bilirubin; BUN: Blood urea nitrogen

**Table 4:** **Table 4:** Comparison between before and after treatment in group I as regards clinical and U/S findings

		*Group I*					
*Variables*		*Before treatment (n = 15)*		*After treatment (n = 15)*		*p-value*	
Clinical presentation							
Asymptomatic		8 (53.3%)		12 (80%)		0.125	
Dyspepsia		4 (26.7%)		1 (6.7%)		0.250	
Fatigue		2 (13.3%)		1 (6.7%)		1.000	
Right hypochondrial pain		1 (6.7%)		1 (6.7%)		1.000	
Clinical examination							
Hepatomegaly		4 (26.7%)		4 (26.7%)		1.000	
Splenomegaly		0 (0%)		0 (0%)		1.000	
Ascites		0 (0%)		0 (0%)		1.000	
Liver in U/S							
Average		4 (26.7%)		4 (33.3%)		0.135	
Enlarged		11 (73.3%)		11 (73.3%)			
Spleen in U/S							
Average		15 (100%)		15 (100%)		1.000	
Splenomegaly		0 (0%)		0 (0%)			
Ascites in U/S							
Absent		15 (100%)		15 (100%)		1.000	
Present		0 (0%)		0 (0%)			

**Table 5:** **Table 5:** Comparison between before and after treatment in group I as regards laboratory findings

		*Group I*					
*Variables*		*Before treatment (n = 15)*		*After treatment (n = 15)*		*p-value*	
Hemoglobin (g/dL)		13.16 ± 0.88		13.20 ± 0.85		0.334	
WBCs (× 10^3^/mm^3^)		6.78 ± 1.72		6.79 ± 1.70		0.806	
Platelet count (× 10^3^/mm^3^)		300.60 ± 78.33		298.40 ± 76.37		0.021	
Protein (g/dL)		7.47 ± 0.77		7.47 ± 0.77		1.000	
Albumin (g/dL)		4.60 ± 0.44		4.58 ± 0.45		0.208	
TSB (mg/dL)		0.88 ± 0.38		0.87 ± 0.37		0.144	
DSB (mg/dL)		0.11 ± 0.04		0.11 ± 0.04		0.334	
ALT (U/L)		83.33 ± 10.96		46.10 ± 20.75		<0.001	
AST (U/L)		57.06 ± 7.86		38.20 ± 11.58		0.03	
Serum creatinine (mg/dL)		0.90 ± 0.29		0.89 ± 0.26		0.414	
BUN (mg/dL)		18.06 ± 6.81		17.73 ± 6.13		0.173	

WBC: While blood cell; TSB: Total serum bilirubin; DSB: Direct serum bilirubin; BUN: Blood urea nitrogen

**Table 6:** **Table 6:** Comparison between before and after treatment in group II as regards laboratory findings

		*Group I*					
*Variables*		*Before treatment (n = 15)*		*After treatment (n = 15)*		*p-value*	
Hemoglobin (g/dL)		13.57 ± 0.84		13.67 ± 0.71		0.355	
WBCs (× 10^3^/mm^3^)		7.36 ± 1.85		7.36 ± 1.67		0.937	
Platelet count (× 10^3^/mm^3^)		312.20 ± 75.56		309.26 ± 70.02		0.366	
Protein (g/dL)		7.45 ± 0.89		7.42 ± 0.80		0.362	
Albumin (g/dL)		4.64 ± 0.45		4.66 ± 0.41		0.519	
TSB (mg/dL)		0.86 ± 0.41		0.88 ± 0.37		0.340	
DSB (mg/dL)		0.12 ± 0.05		0.12 ± 0.05		0.582	
ALT (U/L)		83.53 ± 12.01		82.53 ± 10.41		0.096	
AST (U/L)		58.73 ± 9.95		55.86 ± 5.11		0.048	
Serum creatinine (mg/dL)		1.05 ± 0.30		1.07 ± 0.20		0.638	
BUN (mg/dL)		19.13 ± 7.46		18.46 ± 6.45		0.329	

WBC: While blood cell; TSB: Total serum bilirubin; DSB: Direct serum bilirubin; BUN: Blood urea nitrogen

This study showed improvement of dyspepsia in group I posttreatment; otherwise, there were no significant change in other symptoms and in abdominal U/S findings.

This study also showed significant decrease in ALT and AST in group I posttreatment in comparison to pre-treatment; otherwise, there was no significant change in other laboratory findings, and these were in agreement with the findings of Loguercio et al^21^ and Wong et al.^23^

## CONCLUSION

Probiotics treatment is effective, safe, well-tolerated, inexpensive, appropriate for long-term use, and optimally works at multiple levels to downregulate inflammatory mediators, and therefore, probiotics could be an option in the treatment of NASH.

## References

[1] Cheung O, Sanyal AJ (2010). Recent advances in nonalcoholic fatty liver disease. Curr Opin Gastroenterol.

[2] Musso G, Cassader M, De Michieli F, Rosina F, Orlandi F, Gambino R (2012). Nonalcoholic steatohepatitis versus steatosis: adipose tissue insulin resistance and dysfunctional response to fat ingestion predict liver injury and altered glucose and lipoprotein metabolism. Hepatology.

[3] Day CP, Saksena S (2002). Non-alcoholic steatohepatitis: definitions and pathogenesis. J Gastroenterol Hepatol.

[4] Bugianesi E, Leone N, Vanni E, Marchesini G, Brunello F, Carucci P, Musso A, De Paolis P, Capussotti L, Salizzoni M (2002). Expanding the natural history of nonalcoholic steatohepatitis: from cryptogenic cirrhosis to hepatocellular carcinoma. Gastroenterology.

[5] Medina J, Fernández-Salazar LI, Garcia-Buey L, Moreno-Otero R (2004). Approach to the pathogenesis and treatment of nonalcoholic steatohepatitis. Diabetes Care.

[6] Marchesini G, Brizi M, Morselli-Labate AM, Bianchi G, Bugianesi E, McCullough AJ, Forlani G, Melchionda N (1999). Association of nonalcoholic fatty liver disease with insulin resistance. Am J Med.

[7] Younossi ZM, Diehl AM, Ong JP (2002). Nonalcoholic fatty liver disease: an agenda for clinical research. Hepatology.

[8] Tilg H, Diehl AM (2000). Cytokines in alcoholic and nonalcoholic steatohepatitis. N Engl J Med.

[9] Valenti L, Fracanzani AL, Dongiovanni P, Santorelli G, Branchi A, Taioli E, Fiorelli G, Fargion S (2002). Tumor necrosis factor alpha promoter polymorphisms and insulin resistance in nonalcoholic fatty liver disease. Gastroenterology.

[10] Hoek JB (1999). Endotoxin and alcoholic liver disease: tolerance and susceptibility. Hepatology.

[11] Adachi Y, Moore LE, Bradford BU, Gao W, Thurman RG (1995). Antibiotics prevent liver injury in rats following long-term exposure to ethanol. Gastroenterology.

[12] Nanji AA, Khettry U, Sadrzadeh SM (1994). Lactobacillus feeding reduces endotoxemia and severity of experimental alcoholic liver (disease). Proc Soc Exp Biol Med.

[13] Rolfe RD (2000). The role of probiotic cultures in the control of gastrointestinal health. J Nutr.

[14] Urao M, Fujimoto T, Lane GJ, Seo G, Miyano T (1999). Does probiotics administration decrease serum endotoxin levels in infants?. J Pediatr Surg.

[15] Kumar P., Clark M. (2002). Clinical medicine (textbook)..

[16] Clark JM, Brancati FL, Diehl AM (2003). The prevalence and etiology of elevated aminotransferase levels in the United States. Am J Gastroenterol.

[17] Baldridge AD, Perez-Atayde AR, Graeme-Cook F, Higgins L, Lavine JE (1995). Idiopathic steatohepatitis in childhood: a multi-center retrospective study. J Pediatr.

[18] Neuschwander-Tetri BA (2007). Fatty liver and the metabolic syndrome. Curr Opin Gastroenterol.

[19] Tominaga K, Kurata JH, Chen YK, Fujimoto E, Miyagawa S, Abe I, Kusano Y (1995). Prevalence of fatty liver in Japanese children and relationship to obesity. An epidemiological ultrasonographic survey. Dig Dis Sci.

[20] Solga SF, Diehl AM (2003). Non-alcoholic Fatty liver disease: lumen-liver interactions and possible role for probiotics. J Hepatol.

[21] Loguercio C, Federico A, Tuccillo C, Terracciano F, D’Auria MV, De Simone C, Del Vecchio Blanco C (2005). Beneficial effects of a probiotic VSL#3 on parameters of liver dysfunction in chronic liver diseases. J Clin Gastroenterol.

[22] Portincasa P, Grattagliano I, Palmieri VO, Palasciano G (2006). Current pharmacological treatment of nonalcoholic fatty liver. Curr Med Chem.

[23] Wong VW, Won GL, Chim AM, Chu WC, Yeung DK, Li KC, Chan HL (2013). Treatment of nonalcoholic steatohepatitis with probiotics. A proof-of-concept study. Ann Hepatol.

